# Implementation of Cognitive Behavioral Therapy in e–Mental Health Apps: Literature Review

**DOI:** 10.2196/27791

**Published:** 2022-03-10

**Authors:** Kerstin Denecke, Nicole Schmid, Stephan Nüssli

**Affiliations:** 1 Institute for Medical Informatics Bern University of Applied Sciences Biel Switzerland; 2 Suchtfachklinik Zurich Zurich Switzerland

**Keywords:** cognitive behavioral therapy, mHealth, e–mental health, chatbot, mobile phone

## Abstract

**Background:**

To address the matter of limited resources for treating individuals with mental disorders, e–mental health has gained interest in recent years. More specifically, mobile health (mHealth) apps have been suggested as electronic mental health interventions accompanied by cognitive behavioral therapy (CBT).

**Objective:**

This study aims to identify the therapeutic aspects of CBT that have been implemented in existing mHealth apps and the technologies used. From these, we aim to derive research gaps that should be addressed in the future.

**Methods:**

Three databases were screened for studies on mHealth apps in the context of mental disorders that implement techniques of CBT: PubMed, IEEE Xplore, and ACM Digital Library. The studies were independently selected by 2 reviewers, who then extracted data from the included studies. Data on CBT techniques and their technical implementation in mHealth apps were synthesized narratively.

**Results:**

Of the 530 retrieved citations, 34 (6.4%) studies were included in this review. mHealth apps for CBT exploit two groups of technologies: technologies that implement CBT techniques for cognitive restructuring, behavioral activation, and problem solving (exposure is not yet realized in mHealth apps) and technologies that aim to increase user experience, adherence, and engagement. The synergy of these technologies enables patients to self-manage and self-monitor their mental state and access relevant information on their mental illness, which helps them cope with mental health problems and allows self-treatment.

**Conclusions:**

There are CBT techniques that can be implemented in mHealth apps. Additional research is needed on the efficacy of the mHealth interventions and their side effects, including inequalities because of the digital divide, addictive internet behavior, lack of trust in mHealth, anonymity issues, risks and biases for user groups and social contexts, and ethical implications. Further research is also required to integrate and test psychological theories to improve the impact of mHealth and adherence to the e–mental health interventions.

## Introduction

### Background

Mental disorders, including depressive and anxiety disorders, affect 29% of the global population in their lives [1]. Apart from the fact that mental disorders have an impact on people’s quality of life, they are one of the most common causes of occupational disability [2], resulting in high economic costs. The negative social aspects experienced by individuals with mental disorders include the inability to create and maintain lasting relationships and the stigmatization in society. These factors hamper individuals’ capacity to act and lead a self-determined life as members of society, discourage them from seeking professional help, and possibly reinforce the characteristics of mental disorders [3]. Mental disorders are usually treated using pharmacotherapy or psychotherapy [4]. However, there is a global shortage of mental health professionals as demand exceeds service provision. There are 9 psychiatrists per 100,000 people available in high-income countries [5], whereas there is 1 psychiatrist for every 10 million people in low-income countries [6]. In Europe, a comparative study between Finland and Spain—both with a similar prevalence of mental health disorders—showed a significant difference in the number of available staff resources. In Finland, for example, 13 psychologists were available per 100,000 inhabitants, whereas in Spain, only 2.9 psychologists were available per 100,000 inhabitants [7]. According to the World Health Organization (WHO), approximately 45% of people in high-income countries and 15% of people in low-income countries can access mental health services [8]. Leaving people with untreated mental disorders may increase suicide attempts and mortality [9]. Even if treated, approximately 98% of patients’ change processes induced by therapy occur outside of therapy sessions in their daily lives. Therefore, there is a need to provide support and self-help between therapy sessions, which increases the availability of cognitive behavioral therapy (CBT) to larger populations.

To address the issue of limited resources for treating individuals with mental disorders, e–mental health has gained interest in recent years, particularly for behavior change using elements of CBT and self-help (eg, MoodGym [10]). e–mental health is defined as “mental health services and information delivered or enhanced through the Internet and related technologies.” [11]. Numerous studies have shown that e–mental health interventions are comparable in effectiveness to traditional face-to-face psychotherapy [12,13], thus providing a possible solution for people who do not have access to face-to-face therapy. e–Mental health enables users to learn more about their mental health condition through self-help services; it empowers them to strengthen their self-management and improve their health, sometimes including peer-to-peer support [14]. e–Mental health, realized as mobile health (mHealth) apps, aims to expand the availability and quality of mental health treatment. mHealth apps often ask users to enter data for reflection and awareness and provide relevant information depending on user input. Sometimes, they also collect data from wearables. The number of apps addressing mental health has rapidly increased in recent years [15,16]. Details on the technical implementation of mHealth apps are rarely described in scientific papers [17], although implementation is of utmost importance to enable patient agency and facilitate self-therapy practices. This study aims to investigate which CBT techniques are implemented by which technologies in mHealth apps and derive the research gaps.

### CBT and mHealth Apps

CBT is an “active, problem-focused, and time-sensitive approach to treatment that aims to reduce emotional distress and increase adaptive behavior in patients with a host of mental health and adjustment problems” [15,18]. There are four fundamental techniques of psychotherapy used in CBT: cognitive restructuring, behavioral activation, exposure, and problem solving [15,18]. In cognitive restructuring, therapists support patients in recognizing, evaluating, and modifying maladaptive or unhelpful thinking. Behavioral activation helps patients to actively re-engage in their lives. Exposure comprises systematic contact with a feared stimulus, whereas problem solving aims to help patients identify and implement solutions to their problems. We based our work on the implementation of CBT in mHealth apps on these 4 fundamental techniques of psychotherapy.

The efficacy of CBT has been demonstrated in multiple forms of psychopathology, including anxiety disorders, depression, and eating disorders [19]. Efficacy in the context of this study means that the effectiveness of an intervention can be demonstrated. mHealth apps provide options for practices, which were formerly elements of therapist–patient interaction; thus, this provides momentum for new routines and social forms [20] of coping with mental health problems.

In this study, we aim to assess which technologies are used in mHealth apps to implement the CBT technique. More specifically, we seek to answer the following research questions: for which mental illnesses have CBT-based apps been in use or tested, which CBT techniques are implemented in mHealth apps, which technologies are used to implement CBT techniques in mHealth apps, and which research gaps exist in mHealth apps for realizing CBT?

## Methods

### Overview

We answered our research questions using a literature search and review. We studied which of the 4 fundamental CBT techniques (cognitive restructuring, problem solving, behavioral activation, and exposure) have been implemented in mHealth apps. Furthermore, we investigated the technologies used for their implementation.

### Search Strategy

The PRISMA (Preferred Reporting Items for Systematic Reviews and Meta-Analyses) criteria guided the conduct and reporting of our literature search [21]; for the PRISMA checklist, refer to Multimedia Appendix 1. The search was conducted between June 6 and June 13, 2020, considering all articles published during the period of 2007 to 2020, as the first iPhone was launched in 2007, establishing the technological basis for mobile apps. A total of 3 databases were consulted to find the relevant papers. Papers included in PubMed were retrieved using the search string described in Textbox 1. As we were also interested in the technical aspects of CBT in mHealth apps, we additionally searched the libraries of IEEE Xplore and ACM Digital Library (Textbox 1).

**Search strings used for database search.**

**Search strings used to search PubMed**

- *Cognitive behavioral therapy* AND *mental health* AND (*telemedicine* OR*mobile health* OR mhealth OR smartphone) NOT (*internet delivered* OR *internet-delivered*): 287 results with abstract

**Search strings used to search IEEE Xplore**

- *Cognitive behavioral therapy*: 65 results

**Search strings used to search ACM Digital Library**

- Query: (Abstract: *cognitive behavioral therapy* AND *mental health*) AND (Abstract: *mobile health* OR *mhealth* OR *smartphone*); Filter: (Article type: *Research Article*, Publication date: [1/1/2007 TO *], ACM content: *DL*): 178 results

### Inclusion and Exclusion Criteria

Articles were included in this review if they were dealing with CBT and mHealth apps, they were primary studies reporting results, and adults (aged >18 years) were the target population.

Articles were excluded if the target populations for interventions were military veterans, children, or adolescents. These target groups differ from the general public. Military veterans have a significantly higher prevalence of posttraumatic stress disorder than the average population [22] and often receive care in specialized institutions. On the other hand, digital resources for children or adolescents need to address specific cognitive and developmental issues [23] and cannot be directly compared with apps for adults. Therefore, we decided to exclude apps that explicitly targeted these groups from this review. Furthermore, all papers dealing only with web-based interventions and those describing only the study protocol without the final results were excluded.

### Eligibility and Data Extraction

To assess the eligibility of the articles, all titles and abstracts were examined by 2 independent reviewers (KD and SN) in the first round. In the second round, the full texts of the selected articles were extracted and carefully analyzed to confirm their eligibility. Eligibility doubts were discussed until an agreement was reached. The selected articles were included in the qualitative synthesis.

Two reviewers (KD, NS) extracted data from the selected studies regarding CBT techniques, technologies implemented in the mHealth app, type of mHealth app, considered medical conditions, and outcome. With *CBT techniques*, we referred to the 4 fundamental techniques of psychotherapy applied within CBT (cognitive restructuring, behavioral activation, exposure, and problem solving [15]). With *technologies* in mHealth apps, we implied technical means used in the apps for realizing specific functionalities. More specifically, we assessed the provision of audio and video content, interactive elements (eg, communication facilities with humans or computer systems such as chatbots), social network technologies, gamification, and automatic analysis facilities (eg, for sentiment or emotion detection, recommendation, and text analysis). Data were abstracted into a spreadsheet standardized for this review. Finally, we derived the research gaps in e–mental health from the results.

## Results

### Sample

**Figure 1:**
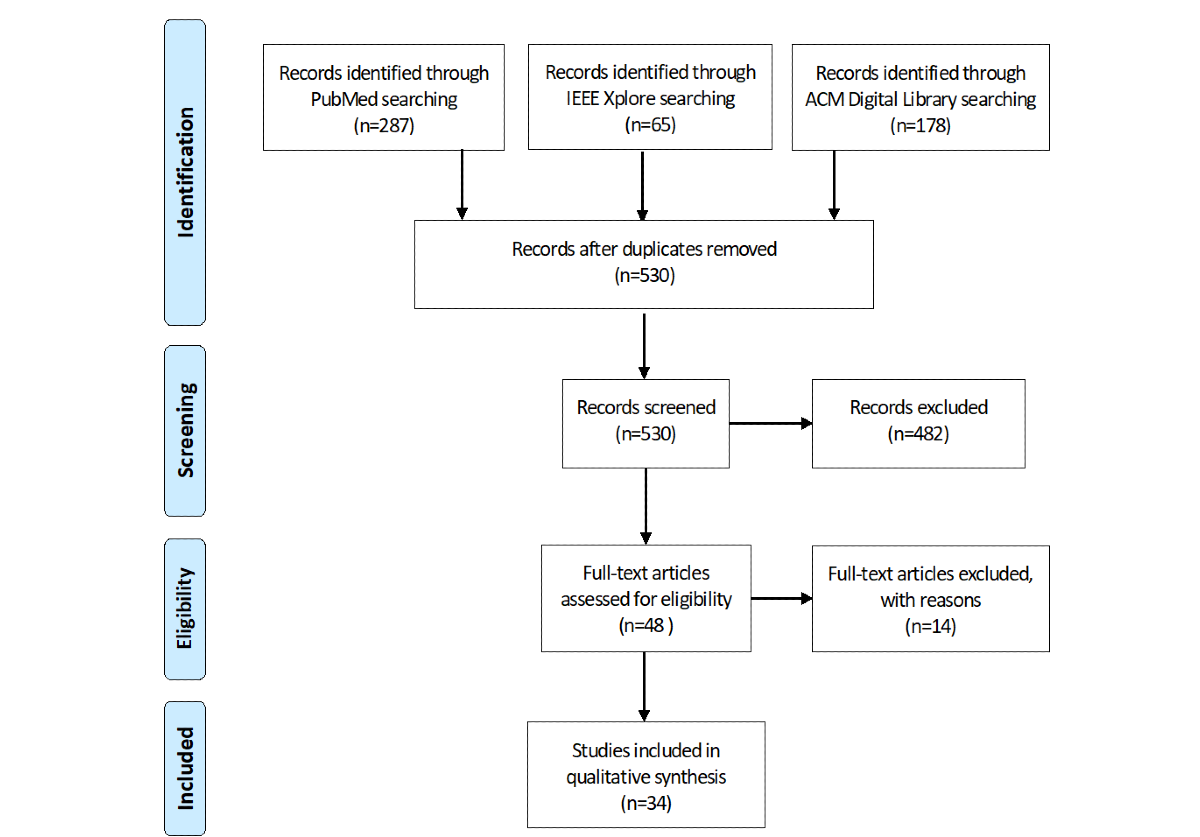
PRISMA (Preferred Reporting Items for Systematic Reviews and Meta-Analyses) flowchart of the selection procedure.

A total of 530 papers were retrieved by our search, as follows: 287 (54.2%) records in PubMed, 65 (12.3%) in IEEE Xplore, 178 (33.6%) in ACM Digital Library, and no duplicates. Of these 530 papers, 34 (6.4%) papers met the inclusion criteria and were, therefore, included in the qualitative synthesis (see the flowchart of the selection procedure in Figure 1). Papers were excluded during eligibility screening and data extraction if they described only mock-ups or no apps.

### Characteristics of Studies

Most papers (21/34, 62%) reported randomized controlled trials (RCTs) in which ≥1 mental health app was tested (Multimedia Appendix 2). The remaining papers used different study designs. Of the 34 papers, 4 (12%) only assessed usability through surveys. For RCTs, the number of involved participants varied between 30 and 1098, with an average of 333. The studies designed as surveys involved an average of 135 participants. We found the largest number of participants (n=3977) in an observational study [24]. This study did not recruit participants explicitly but analyzed the use protocols of the app under consideration. It remains unclear whether these protocols originated from people with diagnosed mental health problems.

Most studies considered usability [25,26], user satisfaction, acceptance [24], adherence [27], or engagement as outcome measures. RCTs mainly studied efficacy, adherence, or acceptability compared with a control group. In RCTs, e–mental health interventions were often compared against each other instead of comparing the app intervention with standard face-to-face psychotherapy. For example, the efficacy of a web-based implementation of the Kokoro app was compared with that of an app-based intervention [28]. Among other things, outcome measures included the Patient Health Questionnaire–9 or Emotional Self-awareness Scale [29]. Of the 34 papers, 3 (9%) studied the Kokoro app for depression. In these papers, the effectiveness of the app-based intervention was reported; the use of the app reduced the symptoms of depression [28,30,31].

### Mental Health Conditions Covered

Most studies (32/34, 94%) focused their research on a user group with a specific disorder. We classified the disorders according to the WHO International Classification of Diseases–10th revision (version 2019) coding system (Table 1). Most studies (16/34, 47%) used apps that offered support for persons with depression. Furthermore, there was a wide range of apps that targeted anxiety disorders; addiction problems; and psychiatric disorders such as schizophrenia, bipolar disorders, or suicidal tendencies. These disorders are often treated by CBT, which can be realized in an mHealth app in contrast to other types of therapies.

**Table 1:** Health conditions examined in the studies (N=34).

Health condition	WHO^a^ ICD-10^b^ code	Total, n (%)
Drug addiction—cannabis	F12.1	1 (3)
Agoraphobia	F40.0	1 (3)
Borderline personality	F60.3	1 (3)
Depression	F32	16 (47)
Eating disorder	F50	2 (6)
Insomnia	G47	1 (3)
Psychosis	F29	2 (6)
Schizophrenia, schizoaffective, or bipolar 1	F20, F25, and F31	1 (3)
Smoking	Z72.0	3 (9)
Stress	F99	3 (9)
Suicidality	R45.8	1 (3)
Unknown	N/A^c^	2 (6)

^a^WHO: World Health Organization.

^b^ICD-10: International Classification of Diseases–10th revision.

^c^N/A: not applicable.

### CBT Techniques in mHealth Apps

Apps may have integrated techniques to address different purposes. We classified the main purposes of the apps as informational, coach, or therapy. An app was considered informational when it mainly provided information to the user, for example, on mental health. Apps were classified as coaches when suggestions were tailored to the user and their specific mental health condition or when support in managing a mental illness was delivered. As therapeutic apps, we grouped apps that delivered CBT with the aim of creating a therapeutic setting.

We could not identify any apps that were only informational. Most apps provided information but had additional functionalities that led to their classification as coach or therapy. Most (20/34, 59%) apps could be considered as coaches in mental health. Of the 34 apps, 13 (38%) were classified as therapy; for 1 (3%) app, this classification was not applicable. We concluded that CBT-based mHealth apps go beyond patient education. They support and extend their self-help capabilities.

We identified in the included papers 3 of the 4 fundamental techniques of psychotherapy applied in CBT (cognitive restructuring, behavioral activation, exposure, and problem solving [15]; Multimedia Appendix 3 [24-27,29,30,32-50]). mHealth apps provided toolkits for cognitive restructuring, which included diary-keeping functionalities and support in changing thoughts or tensions. Behavioral activation was realized by providing information on mental health conditions by tracking activities or setting goals. Assigning homework to patients, which is the best practice as a problem-solving technique [15], was realized in many apps (20/34, 59%) by providing exercises and activities as strategies to cope with mental health problems. Direct communication with a therapist, which is not a CBT technique itself but helps in standard setting for delivering CBT, was enabled by 6% (2/34) of the apps included in the review.

### Technologies in mHealth Apps for Implementing CBT

When developing mHealth apps for CBT, it is important to know which technologies are useful and efficient for implementing different CBT techniques. Interactive elements were included in 35% (12/34) of apps to realize behavioral activation and cognitive restructuring. Among other things, we could identify the following interactive elements: automatic question answering functionalities, message exchange with the treatment team, a virtual character that provided information or explanations, personalization, and persuasion methods (Multimedia Appendix 4 [24-28,31-37,40-46,50-54]). Of the 34 apps, 3 (8%) provided a conversational agent or chatbot with which the user could communicate. Chatbot technology was used to support cognitive restructuring. Methods for automatic analysis of free textual input (natural language processing) and machine learning methods (eg, text classification or clustering) were used to understand user input or personalize recommendations according to user input. However, these methods are still rarely used (3/34, 9% papers). Audio and video were used to provide information or demonstrate exercises such as meditation exercises (ie, support behavioral activation and problem solving). Few apps calculated scores such as sentiment or emotion scores that were shown on a timeline. Sentiment or emotion scores quantify the sentiment or emotion of a user (eg, positive and negative sentiment or the strength of an emotion such as sadness). The following two technologies were integrated into some apps that aimed to improve the user experience and adherence: gamification and social networks. Social aspects were integrated, enabling the user to connect with other users. This social community aspect was interesting in the context of depression but also in other mental diseases, where people often experience loneliness and a lack of social contact. Gamification and social networks integrated into these apps did not aim to deliver CBT but, more importantly, aimed to increase the adherence and attractiveness of using the apps [32,51]. Gamification such as collecting jigsaws was used in 24% (8/34) of apps, and audio or video content was provided by 21% (7/34) of apps. Approximately 15% (5/34) of apps enabled connections with social networks and communities.

## Discussion

### Principal Findings

#### Overview

The main finding of this review is that mHealth apps for CBT exploit two groups of technologies: (1) technologies that implement concrete CBT techniques for cognitive restructuring, behavioral activation, and problem solving and (2) technologies that aim to increase user experience, adherence, and engagement. The latter tries to address the current challenge in delivering CBT, which is insufficient adherence to CBT techniques [55]. A CBT technique that we did not find implemented was *exposure*. There is a broad range of technologies used in mHealth apps to deliver CBT; however, no technology was used in all apps. However, a trend toward the use of interactive elements, gamification technologies, and technologies supporting social activities could be recognized. No app was purely informational; however, most could be classified as coaches and a few as therapeutic. There have been attempts to deliver therapy through such apps [33-35,52]. Most apps target patients with disorders that are often treated with CBT, such as depression; anxiety disorders; addiction problems; and psychiatric disorders such as schizophrenia, bipolar disorders, or suicidal tendencies.

#### Mental Illnesses for Which CBT-Based Apps Have Been Used or Tested

Depression was reported most often in the assessed studies. Depressive disorders are widespread in all countries and comorbid with other mental diseases. According to the WHO, >264 million people of all ages experience depression worldwide. Thus, the target user group is huge. Depression is a leading cause of disability worldwide and a major contributor to the overall global burden of disease. There seems to be a burden of being able to deliver support to those people, and mental health apps are obviously comprehensively tested to fill this gap. Although our review did not deliver much evidence on the efficacy of mHealth apps in the mental health context, Khademian et al [56] found that mHealth apps that provide behavior change strategies, such as CBT and techniques for behavioral activation, have significant effects on depression, anxiety, and stress.

Psychiatric diseases such as psychosis or schizophrenia were also targeted. A challenge with psychiatric diseases is that critical situations can occur where professional reactions are essential (eg, to prevent a suicide attempt [57]). mHealth apps that deliver predefined content cannot react individually to various situations or specific user needs. There is a need for mHealth apps without undesired side effects; that is, those able to respond appropriately in situations of crisis [17].

#### CBT Techniques Implemented in mHealth Apps

We found that some methods used in standard CBT were implemented in mHealth apps. Sometimes, it was simply a digitization of the existing technique; however, there were cases where mHealth apps offered some benefits compared with the traditional therapy setting. Thought records or coping cards are methods used within standard CBT to achieve cognitive restructuring [15]. These methods are often provided in mHealth apps, for example, by supporting the keeping of diaries.

Scheduling and monitoring activities are the central components of behavioral activation in CBT [58]. Behavioral activation recommends planning activities in the evening before or in the early morning. As therapy sessions are spread across weeks and planning tasks are conducted with the therapist, day-to-day planning is not practiced in traditional therapy settings. With the use of mHealth technologies, daily planning and engagement can be supported, and personalized recommendations can be made [36]. Furthermore, increasing patients’ confidence in their ability to cope with stress and adversity and their overall coping skills is a major factor in mHealth apps contributing to the effects on mental health [29].

Information provision as a means of behavioral activation is another important aspect realized in many apps. This task includes teaching patients the basics of their mental health condition and CBT techniques. Morriss et al [59,60] have shown that knowledge and understanding of the medical condition are effective in supporting the everyday coping of patients with mental illness and can foster compliance of patients to the health intervention. Furthermore, patients become more competent in making decisions related to their health through information provision [61]. As providing information is a repetitive task for health care providers, an app providing information and teaching patients on the basics of mental health could save time for health care providers. Furthermore, the patient can read the information several times. However, studies show that e–mental health apps often contain incorrect information [62] and are not necessarily evidence based.

Assigning homework to patients is the best practice as a problem solving technique [15]. This aspect of CBT, which is available in many apps, is realized by providing exercises and activities. Exposure (imaginal exposure and interoceptive exposure [15]) is a CBT technique that was not represented at all in mental health apps included in our review. However, recent research has exploited virtual reality for realizing exposure apps; for example, to reduce the fear of heights [63]. It is obvious that there is still potential for developing and testing mHealth apps that target exposure techniques.

In conclusion, mental health apps that provide CBT support the performing of repetitive tasks such as keeping a diary or exercising, which are essential tasks and techniques within CBT for cognitive restructuring, behavioral activation, and problem solving [15]. Although these tasks must be realized by patients as part of their standard therapy, even without mHealth support, their digitization in mHealth apps can increase adherence, user engagement, and retention or facilitate learning [64]. A mobile app is available 24 hours a day. Thus, monitoring and tracking can be performed at any time and at any place. Records in the app cannot get lost as paper-based records do. Reminders can be sent to the user on a regular basis to ensure that records are made or activities come into the user’s mind [37].

#### Technologies Used to Implement CBT in mHealth Apps

Most apps often present information using videos or audio, or they collect data on activities from a user for behavioral activation. Social aspects are integrated, enabling the user to connect with other users. This social community aspect is interesting not only in the context of depression but also other mental diseases, where people often experience loneliness and a lack of social contact. Gamification and social networks integrated in these apps do not aim to deliver CBT but, more importantly, aim to increase the adherence and attractiveness of using these apps [37]. Artificial intelligence, including natural language processing, language understanding, and chatbot technology, has been rarely used in available apps. A reason might be that unforeseen errors or reactions can occur when the system misinterprets the user input, which might be avoided [65]. Other studies have shown an increased interest in mental health chatbots [66] or that chatbots are examples of the next generation of mental health [17]. However, this was not reflected in our review. Another reason for the limited inclusion of artificial intelligence in mental health apps may be related to medical device regulation, which requires traceability and increases the demand on the development process.

#### Research Gaps That Exist in mHealth Apps for Realizing CBT

After scoping the landscape of mHealth apps that implement CBT, we summarized the open research issues. There is a need for the following: RCTs studying the efficacy of the single technologies implemented in mHealth apps for realizing CBT techniques; studies on the impact on patients’ agency, including trust and overreliance; consideration of psychological theories during mHealth implementation to increase impact and adherence; and support in recommending or selecting CBT apps as health interventions.

Although more than half of the analyzed studies reported on RCTs, which is the state-of-the-art study design for proving the efficacy of medical interventions [67], these trials often did not assess the efficacy of the mHealth intervention. Similar results have been reported by Bauer et al [68]. For chatbots in clinical psychology, Bendig et al [17] noted that studies on mental health chatbots mainly assessed feasibility and acceptance. Studies are needed to assess which technology is well-suited for implementing a particular CBT technique, as well as for which mental disorders are mHealth apps efficient. This will help derive the best practices for implementing CBT techniques in mHealth. In this context, it is also important to clarify the role of mHealth apps; for example, whether they are intended to accompany the therapy or fill the treatment gap until a therapy can be started.

We recognize that the impact of mHealth apps on patients’ agency is not explicitly considered in these studies. Agency is generally viewed as the “capacity to act, produce and anticipate a desired outcome within a particular context” [69]. In our context, this means, among other things, that through mental health apps, patients would begin to manage themselves as participants with agency exercised on the basis of patient autonomy [70].

From a technical perspective, we want to achieve good adherence and frequent use of apps and support this by integrating features such as gamification to encourage frequent use. However, there are also concerns about overreliance on mental health apps, particularly for people who already have trouble with addictive web-based behavior [71]. The use of smartphone apps might be problematic, given specific mental conditions such as anxiety or depression [72]. Studies that assess this risk of overreliance in long-term use of mHealth apps, as well as assessments related to trust in mHealth from perspectives of both patients and therapists, are missing. There are indications that users think they have even more trust in an mHealth app when reporting personal issues than in their physician [73]. Anonymity helps overcome thoughts of stigmatization or shame when describing personal issues [74].

We are also missing implementations of psychological theories in CBT mental health apps to increase the impact of and adherence to these technological means. Peters et al [75] suggested a framework grounded in psychological research that could help developers of mHealth apps develop apps that increase motivation and engagement and, in this way, also have an impact on patients’ well-being.

#### Recommendation of Apps

Our literature search showed that the underlying clinical evidence, technical issues, and implementation details are rarely described in the published research on mental health apps. Without information on the correctness of the underlying evidence base and without confirmation of efficacy, it is challenging for patients to identify the mental health apps that are suited for them.

Therapists and patients rely on lists of *top mental health apps* for their decision-making process, which are not helpful unless they list their ranking and selection criteria for creating the list. We checked for 21 apps listed on the mental health app ranking lists to determine whether there were publications available. A scientific paper or results from a clinical trial were available for only 29% (6/21) of the apps. This makes it difficult for users and health professionals to identify high-quality, evidence-based apps. It must be ensured that the underlying principles and delivered contents are evidence based [17,76] to avoid harm to users, as well as undesired consequences for the users. The number of downloads from an app store is clearly not an indicator of high-quality mHealth apps. On the other hand, we found studies on 28 apps, of which only 16 (57%) were implemented for the established Android and iOS operating systems. Of these 16 apps, only 12 (75%) were available in the stores. This means that apps that have been thoroughly investigated are not available to a wide range of users.

We conclude that there is a need to enable patients in informed decision-making when selecting an mHealth app for CBT [77]. We suggest a minimal data set of information on a mental health app that includes the following items: underlying clinical evidence and CBT techniques that are integrated; information on how CBT techniques are implemented in the app, that is, which technologies are used and for which purpose; efficacy from an RCT, if available; intended application areas of the app; possible contraindications; information on data storage, data security, and privacy; and the integrated third-party tools.

Standardized evaluation metrics should be developed to ensure that only high-quality apps are recommended by therapists. Attempts have been made to harmonize technical metrics for evaluating health chatbots [78,79]. Existing evaluation frameworks for mHealth apps target usability [76]. Although good usability is essential to make sure that users can interact with the app, it is highly relevant that the app really positively affects patients and does not harm them.

### Strengths and Limitations of Our Presented Research Work

#### Strengths

This literature review was conducted to identify the technologies used in mHealth apps for delivering CBT and identify the research gaps. To the best of our knowledge, this study is among the first to summarize these aspects and provide an overview. This study helps in identifying the opportunities and limitations of mHealth in supporting CBT. It also shows the potential and limitations of e–mental health with respect to the self-help capabilities of patients.

As 2 reviewers independently selected the articles and extracted the data, selection bias in this review was reduced. There is a review of Stawarz et al [80] that studies user experience of CBT-enabling apps [80]. In contrast to their work, we are more interested in the technical implementation of CBT in mHealth apps and the research gaps and not only in the features provided by the apps.

#### Limitations

The search in this review was restricted to English and German articles. Accordingly, it is likely that this review missed some publications. We also missed apps for which no publications were available. In addition, we restricted the search to papers that included the terms *cognitive behavior therapy* or *mHealth* or the respective Medical Subject Headings terms. We might have missed relevant papers that did not explicitly use these terms but dealt with a relevant topic. Several terms can be related to mHealth; however, these are too broad to be reviewed in a reasonable amount of time.

### Conclusions

In this paper, we studied the therapeutic aspects of CBT that are implemented in mHealth apps and the technologies by which they are integrated into these apps. We conclude that some CBT techniques (behavioral activation and cognitive restructuring) can be well-realized in a mental health app, whereas others are more difficult to implement (problem solving and exposure). mHealth apps for CBT can support patients through additional self-help and self-management tools that support specific aspects of treatment. mHealth apps facilitate the self-competence and self‑management of patients in coping with mental health problems. Interactive elements, gamification, and integration of social networks are technologies that increase user engagement and adherence. In this way, patients are provided with additional action and interaction capabilities. As they cover relevant aspects of CBT treatment, mHealth apps can, in principle, alleviate the issue of a global shortage of mental health human resources, as they are usually available to millions of users anytime and anywhere, presuming the users have mobile devices and internet access. This, in turn, has the potential to improve the availability and quality of mental health care at reduced expenses. However, these apps must be evidence based and reliable to be effective and avoid harm to patients. Further research is needed on the side effects of mHealth self-help treatments, such as new inequalities because of the digital divide, addictive internet behavior, trust in mHealth, anonymity issues, risk and range of self-help treatments, bias toward user groups and social contexts, and ethical implications.

The future role of app-based e–mental health still has to be clarified. Future research should assess integrated care models where all stakeholders can benefit: the health care provider by avoiding repetitive tasks and the patient by receiving support even beyond therapy sessions. In addition, economic value is relevant for bringing evidence-based apps to users and supporting clinical care. For obsessive-compulsive disorders, it has already been shown that computerized CBT delivered as low-intensity interventions is cost-effective [81].

## Appendix

Multimedia Appendix 1 PRISMA (Preferred Reporting Items for Systematic Reviews and Meta-Analyses) checklist.

Multimedia Appendix 2 Study designs reported in the papers.

Multimedia Appendix 3 Cognitive behavioral therapy tools and methods realized in the reported apps.

Multimedia Appendix 4 Technologies used in the apps.

## Abbreviations

CBT: cognitive behavioral therapy
mHealth: mobile health
PRISMA: Preferred Reporting Items for Systematic Reviews and Meta-Analyses
RCT: randomized controlled trial
WHO: World Health Organization
