# Supplementary material for: Implementation of Cognitive Behavioral Therapy in e–Mental Health Apps: Literature Review
Source: J Med Internet Res. 2022 Mar 10;24(3):e27791. doi: 10.2196/27791 (PMC8949700; doi:10.2196/27791)
Supplement: Multimedia Appendix 2 [file jmir_v24i3e27791_app2.docx]

### Appendix 2: Study designs reported in the papers

| **Study design** | **Total number (%)**  **n=34** | **Average number of participants** | **Range of participant numbers** |
| --- | --- | --- | --- |
| RCT | 21 (62%) | 333 | 30-1098 |
| Observational | 2 (6%) | 5 | - |
| Feasibility study | 3 (9%) | 14 | 7-17 |
| Qualitative short-term longitudinal study | 2 (6%) | 17 | 5-29 |
| Pilot study | 1 (3%) | 3977 | - |
| Survey | 4 (12%) | 135 | 16-482 |
| Exploratory study | 1 (3%) | 32 | - |
