# Supplementary material for: Implementation of Cognitive Behavioral Therapy in e–Mental Health Apps: Literature Review
Source: J Med Internet Res. 2022 Mar 10;24(3):e27791. doi: 10.2196/27791 (PMC8949700; doi:10.2196/27791)
Supplement: Multimedia Appendix 3 [file jmir_v24i3e27791_app3.docx]

### Appendix 3: CBT tools and methods realized in the reported applications

| **CBT technique** | **CBT tools and methods** | **Total number (n=34)** | **Per-**  **centage** | **Papers** |
| --- | --- | --- | --- | --- |
| **Cognitive restructuring** | Cognitive modification (e.g., ABC Theory (Ellis 1991), assess and reduce tension, change thoughts...) | 10 | 29% | [25, 27, 30, 33, 35-37, 67-69] |
| **Cognitive restructuring** | Thought diary, taking notes on emotions etc. | 9 | 27% | [25, 29, 37, 49, 68, 69, 71, 72, 73] |
| **Behavioral activation** | Information provision | 11 | 32% | [24, 25, 27, 29, 30, 33, 35, 69, 74, 75, 71,] |
| **Behavioral activation** | Self-assessment | 7 | 20% | [26, 27, 33, 35, 69, 70, 71] |
| **Behavioral activation** | Setting goals | 3 | 9% | [26, 71, 75] |
| **Behavioral activation** | Tracking / monitoring mood or behavior or activities | 8 | 27% | [25, 27, 29, 37, 70, 74, 75, 76] |
| **Problem solving** | Providing activities and/or exercises as homeworks | 20 | 59% | [25, 26, 27, 29, 33, 35, 36, 37, 42, 69, 70, 71, 73, 74, 75, 76, 77, 78, 79] |
| not applicable | Direct communication with therapist | 2 | 6% | [36, 72] |
