# Supplementary material for: Implementation of Cognitive Behavioral Therapy in e–Mental Health Apps: Literature Review
Source: J Med Internet Res. 2022 Mar 10;24(3):e27791. doi: 10.2196/27791 (PMC8949700; doi:10.2196/27791)
Supplement: Multimedia Appendix 4 [file jmir_v24i3e27791_app4.docx]

### Appendix 4: Technologies used in the applications

| **Technology** | **Total number** | **Papers** |
| --- | --- | --- |
| Calculations | 3 | [25, 26, 33] |
| Chatbot / conversational agents | 3 | [25, 27, 69] |
| Gamification | 8 | [28, 31, 32, 35, 72, 73, 75, 80] |
| Video / audio content | 7 | [24, 25, 37, 69, 71, 73, 79] |
| Social network / community | 5 | [24, 32, 33, 49, 75] |
| Natural language processing / Machine learning | 3 | [25, 42, 70] |
| Interactive elements | 12 | [24, 26, 28, 31, 32, 33, 36, 38, 49, 71, 74, 81] |
